# Mitochondrial DNA: the overlooked oncogenome?

**DOI:** 10.1186/s12915-019-0668-y

**Published:** 2019-07-08

**Authors:** Payam A. Gammage, Christian Frezza

**Affiliations:** 1 MRC Mitochondrial Biology Unit, University of Cambridge, Cambridge, UK; 2 CRUK Beatson Institute for Cancer Research, Glasgow, UK; 3 MRC Cancer Unit, University of Cambridge, Cambridge, UK

**Keywords:** Mitochondria, mtDNA, Cancer, Metabolism

## Abstract

Perturbed mitochondrial bioenergetics constitute a core pillar of cancer-associated metabolic dysfunction. While mitochondrial dysfunction in cancer may result from myriad biochemical causes, a historically neglected source is that of the mitochondrial genome. Recent large-scale sequencing efforts and clinical studies have highlighted the prevalence of mutations in mitochondrial DNA (mtDNA) in human tumours and their potential roles in cancer progression. In this review we discuss the biology of the mitochondrial genome, sources of mtDNA mutations, and experimental evidence of a role for mtDNA mutations in cancer. We also propose a ‘metabolic licensing’ model for mtDNA mutation-derived dysfunction in cancer initiation and progression.

## Mitochondria and metabolism

The mammalian mitochondrion, though primarily of proteobacterial origin, is an evolutionary mosaic composed of elements drawn from and recombined between eukarya, archaea, bacteria, and phage [1–3]. Throughout evolution most mitochondrial genetic information has transferred to the nucleus; however, mitochondria have retained a vestigial genome, mitochondrial DNA (mtDNA), allowing a form of genomic symbiosis through which mitochondria maintain a degree of cellular control, communicating with the nucleus through an incompletely understood series of retrograde signals [4].

Mitochondria are essential organelles for eukaryotes, performing key functions ranging from the generation of bioenergetic intermediates such as ATP and GTP, to the synthesis of nucleotides, Fe-S clusters, haem and amino acids, Fe^2+^/Ca^2+^ handling, inflammation, and apoptosis [5]. By virtue of their position at such a cellular nexus, dysfunction of mitochondria and subsequent metabolic defects are implicated in diverse human pathologies, including both sporadic and familial forms of cancer [6].

Perturbed cellular metabolism in cancerous tissue is an historic and widely recognised phenomenon [7], with recent seminal studies defining specific pathways to mitochondrial dysfunction in cancer through mutation or dysregulated expression of nuclear DNA encoding mitochondrial proteins [8, 9]. More recently, a less-discussed orthogonal route to mitochondrial dysfunction in cancer has been considered: mutation and dysregulation of the mitochondrial genome. In this article, we will review the most recent evidence in support of a role for mtDNA mutations in cancer, the likely source of these mutations, and major challenges that remain to be addressed by the field.

## Genetics of mammalian mitochondria

**Fig. 1.**
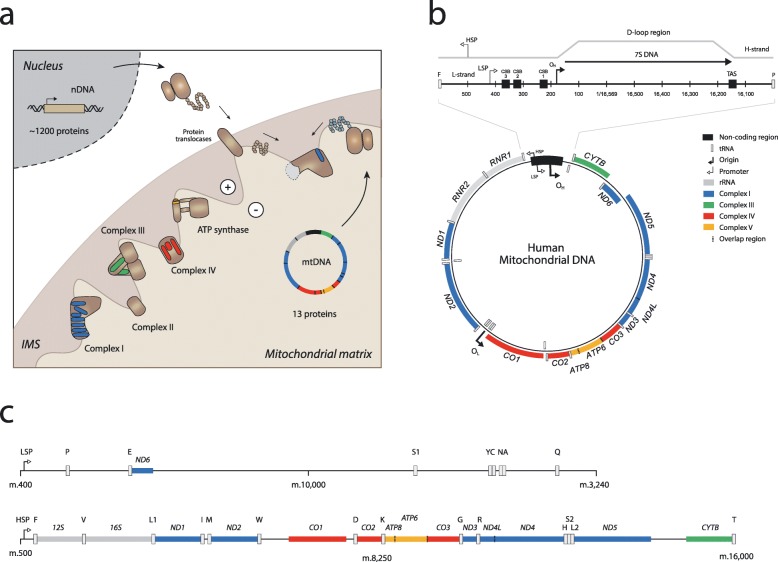
Genetic composition of human mitochondria. **a** Dual-genome origins of the mitochondrial electron transport chain (ETC). The ETC comprises ~ 90 individual protein subunits, encoded by both nuclear (nDNA) and mitochondrial genomes (mtDNA). Assembly of a functional ETC requires co-ordinated regulation and expression of these components by the two separate genomes. Beyond the 13 ETC proteins encoded in mtDNA, the remainder of the human mitochondrial proteome is encoded in and expressed from the nuclear genome. Import of nuclear-encoded proteins through membrane-embedded protein translocases into the mitochondrial matrix requires a membrane potential between the intermembrane space (IMS) and the matrix (white circles). Nuclear encoded components coloured *brown*, mitochondria-encoded components in *blue*, *red*, *green*, and *yellow* by complex. Complex III is shown as a dimer. **b** Annotated genetic features of human mtDNA. Eleven mRNAs (two overlapping) encode 13 polypeptides forming essential components of the ETC. These are expressed using an altered genetic code, enabled by a full complement of 22 mitochondria-specific tRNAs also encoded in mtDNA. Resulting proteins are co-translationally inserted into the inner mitochondrial membrane (IMM) by mitochondrial ribosomes, which contain structural RNA components of exclusive mitochondrial origin (12S rRNA, 16S rRNA, and mt-tRNA^Val^). An expanded view of the displacement loop (D-loop) and major non-coding region (NCR), incorporating 7S DNA, with indication of key loci for mtDNA transcription (heavy strand promoter, *HSP*; light strand promoter, *LSP*), replication (origin of heavy strand, *O*_*H*_) and other prominent elements relevant to these functions (conserved sequence block 1–3, *CSB1–3*; termination-associated sequence, *TAS*). **c** Primary polycistronic maps of transcription of mtDNA from LSP and HSP. Near-complete genome length transcripts are produced through transcription by the mitochondrial RNA polymerase (POLRMT) mitochondrial transcription elongation factor (TEFM) complex, which undergo endonucleolytic processing to liberate individual gene products, and further modifications of mRNA, rRNA, and tRNA molecules to enable efficient translation

The mammalian mitochondrion is formed of ~ 1200 proteins, the vast majority of which are encoded in and expressed from the nuclear genome, whilst a small subset of these proteins is encoded by the spatially and heritably separate mitochondrial genome [10, 11] (Fig. 1a). The human mitochondrial genome is a genetically compact, circular, double-stranded DNA molecule of 16.5 kb, typically present at between 100 and 10,000 copies per cell on a cell type-specific basis [12, 13]. In most higher metazoans, mtDNA is firmly anchored to the inner mitochondrial membrane (IMM) within the mitochondrial matrix, packaged into protein–DNA complexes known as nucleoids, which are formed principally of the mitochondrial transcription factor A (TFAM) [14, 15]. Human mtDNA encodes only 11 mRNAs, 22 tRNAs, and 2 rRNAs [16] (Fig. 1b). In total, 13 extremely hydrophobic polypeptides from these 11 mRNAs are co-translationally inserted into the IMM, where they form core, membrane-bound subunits of respiratory chain complexes I, III, IV, and ATP synthase.

Human mtDNA has an unremarkable GC content (44.4%); however, the biased distribution of these bases across the two strands results in variable buoyancy when mtDNA is resolved using an alkaline caesium chloride gradient, resulting in the G-rich ‘heavy strand’ (H-strand) and C-rich ‘light strand’ (L-strand) nomenclature [17]. An unusual feature of mtDNA is the displacement loop (D-loop), a triple-stranded region of the molecule that incorporates a short single-stranded DNA fragment known as 7S DNA (Fig. 1b). The D-loop is believed to be the product of mtDNA replication events that abort at the termination associated sequence (TAS) within the major non-coding region (NCR). The functional relevance of 7S DNA and the D-loop remains to be fully elucidated (for a thorough review see [18]).

The mitochondrial genome is expressed through transcription by a complex consisting of mitochondrial RNA polymerase (POLRMT) and mitochondrial transcription elongation factor (TEFM) [19] into near genome length polycistrons from either the light-strand promoter (LSP) or the heavy-strand promoter (HSP) (Fig. 1c). Most mRNA-coding genes are separated, or punctuated, by tRNA genes, which are excised from the primary transcript by ELAC2 and mitochondrial RNase P. These molecules are then extensively processed (polyadenylation, various base and sugar modifications [20]), likely within mitochondrial RNA granules, before translation and co-translational insertion of the polypeptides into the IMM by mitochondrial ribosomes [21].

**Fig. 2.**
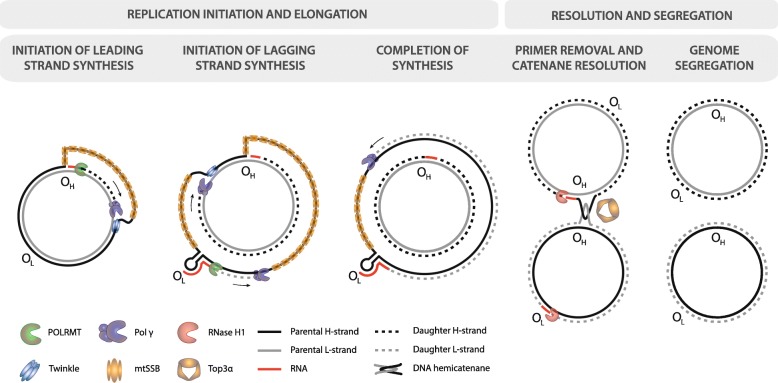
Replication of mtDNA by asynchronous strand-displacement synthesis. Initiation of replication occurs through synthesis of an RNA primer from LSP that forms a G-quadruplex with non-template DNA and terminates at CSB2. The replicative mitochondrial DNA polymerase γ (Pol γ) begins DNA synthesis from this primer around O_H_, with helicase Twinkle unwinding upstream DNA. The parental L-strand acts as the template for synthesis, with the displaced H-strand being temporarily coated in mitochondrial single-stranded binding protein (mtSSB). Once Twinkle reveals O_L_, a stem-loop forms in the ssDNA of the parental H-strand, allowing synthesis of a short RNA primer by POLRMT and subsequent synthesis of the daughter L-strand using the displaced parental H-strand as a template. DNA synthesis proceeds until two complete, hemicatenated mtDNA molecules are produced. RNA primers are removed in a two-nuclease pathway involving RNase H1 and flap endonuclease 1 (FEN1) or FEN1-like activity (not shown), and hemicatenanes are resolved by mitochondrial topoisomerase 3α (*Top3α*)

The replication of mtDNA proceeds in an asynchronous manner through a strand-displacement mechanism, initiated by an RNA primer transcribed by POLRMT from LSP that terminates at a G-quadruplex in nascent RNA and non-template DNA formed at conserved sequence block 2 (CSB2) [22]. The replicative mitochondrial DNA polymerase γ (Pol γ) binds and initiates DNA synthesis from this primer at the origin of the heavy strand (O_H_) within the NCR, located on the L-strand (Fig. 2). The advancing replication fork, consisting of the phage-like Pol γ and helicase Twinkle, synthesises daughter H-strand using L-strand DNA as the template, with the displaced parental H-strand, once unwound by Twinkle, being rapidly coated in mitochondrial single-stranded binding protein (mtSSB) (Fig. 2). The replication fork proceeds and, after ~ 11 kb, the origin of the light strand (O_L_) is revealed in the parent H-strand DNA, forming a stem-loop structure that allows initiation of L-strand synthesis from an RNA primer generated by POLRMT [23]. Once both strands have completed the replicative cycle, RNA primers are removed by RNA:DNA hybrid-specific ribonuclease RNase H1 and Flap endonuclease 1 (FEN1), or FEN1-like activity, with gaps filled and ligated by Pol γ and DNA ligase III, respectively [23]. An unusual, theta-like structure in DNA is formed, with two complete mtDNA molecules linked through a hemicatenated junction near the NCR. Recent data shed light on the segregation of mtDNA following replication, with parent and daughter molecule resolution occurring in a topoisomerase 3α-dependent manner [24]. Some controversy exists concerning the exact sequence of events in mtDNA replication, and particular disagreements on the role of RNA in mtDNA replication, either in the form of Okazaki fragments or as nascent pre-mRNA molecules coating displaced strands, have attracted attention historically [25]. However, the weight of evidence currently favours the classic, asynchronous strand-displacement model [26].

Despite the long-since established status of mtDNA as a multicopy genome with robust copy number control, the basis of any mechanism regulating copy number remains a poorly understood phenomenon [13]. The importance of maintaining mtDNA copy number is also unclear, with several striking examples of total or near-total loss of mtDNA copy number in vitro and in vivo resulting in subtle or temporally delayed effects on mitochondrial function [27, 28]. The multi-copy nature of mtDNA allows for the existence of mixed populations of mtDNA molecules, where not all genomes are identical, a phenomenon known as heteroplasmy. In the disease context, the extent of mtDNA mutation heteroplasmy within a given cell or individual plays an important role in the development of mitochondrial dysfunction, and mitochondrial DNA heterogeneity is an important concept, both in disease and non-disease states [29].

## Source of mtDNA mutations in disease

As with any genetic material, mtDNA is susceptible to damage, errors of nucleic acid metabolism, and imperfect replicative fidelity. Historically, a higher basal mutation rate of mtDNA as compared to nuclear DNA [30] combined with a broadly inferred lack of mtDNA repair from early studies (see for instance [31]) have led to much of the mtDNA mutational burden being ascribed to oxidative damage, specifically from radical oxygen generated by the respiratory chain, and ineffective or absent mtDNA repair mechanisms. That mutations of mtDNA accumulate during ageing and are a common feature of age-related diseases is suggested to further support this view through a ‘vicious cycle’ theory, where greater mutational burden begets a greater oxidative stress, leading to more extensive mutagenesis [32]. Considering our current understanding, however, such a view of mtDNA mutations appears implausible.

While mitochondria lack key nucleotide excision repair (NER) proteins necessary to remove classic bulky DNA adducts (e.g. pyrimidine dimers, cisplatin crosslinks), the existence of both short and long patch base excision repair (BER) and single strand break repair pathways within the mitochondrial compartment have been confirmed [33]. However, mitochondria employ an esoteric strategy for handling of double strand breaks, rapid degradation of the entire genome by components of the replisome [34, 35], that effectively rules out efficient homologous recombination (HR), microhomology-mediated end joining (MMEJ), and non-homologous end joining (NHEJ) [36].

**Fig. 3.**
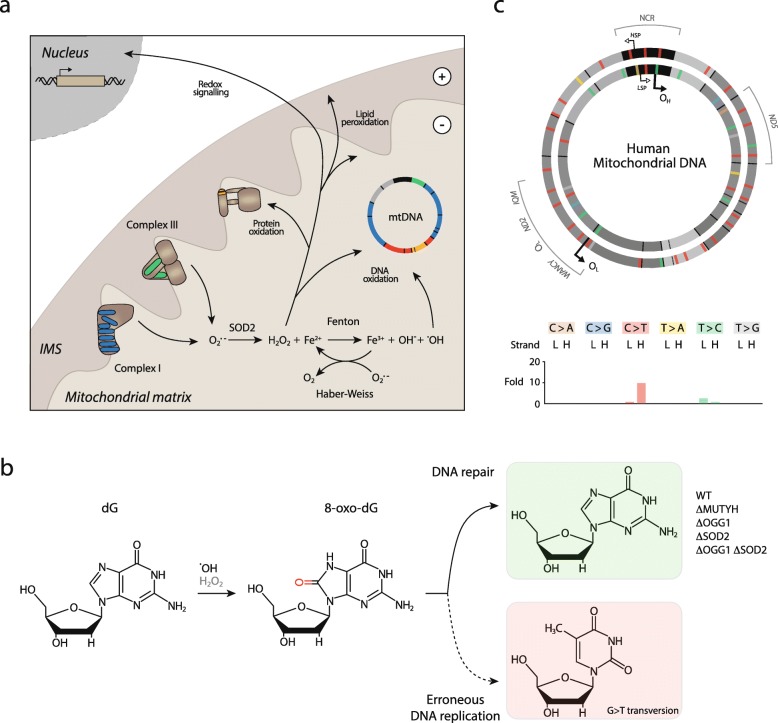
The source and nature of mutations in mtDNA. **a** Simple schematic of radical oxygen generation by the mitochondrial ETC. Superoxide (O_2_^•-^), the proximal mitochondria radical oxygen species, is primarily produced at the flavin mononucleotide site of complex I, and the Q_o_ site of complex III. O_2_^•-^ is rapidly dismutated to hydrogen peroxide (H_2_O_2_) by mitochondrial superoxide dismutase (SOD2). H_2_O_2_ may act as a signalling molecule, but can also introduce oxidative lesions to lipid, protein, and nucleic acid. In the presence of O_2_^•-^ and ferric iron, H_2_O_2_ may also participate in redox cycling Fenton and Haber-Weiss chemistry, producing highly reactive hydroxyl radicals (^•^OH) that present a major oxidative stress to biological systems. **b** Skeletal formula of deoxyguanosine (dG) and its oxidised derivative 8-oxo-guanosine, which can be produced through reaction with either H_2_O_2_ or ^•^OH (phosphates not depicted for clarity). Theoretically, this oxidation should result in G > T mutations following erroneous DNA replication; however, no increase in such mutations is detected in mtDNA following: knockout of individual DNA glycosylases required for repair of this lesion (ΔMUTYH, ΔOGG1), increased oxidative burden (ΔSOD2), or even a double knockout (ΔOGG1, ΔSOD2) in mice [40]. **c** The nature of mtDNA mutations detected in 527 human tumours of varying pathology. Regions and genes within mtDNA that are mutated with higher than expected occurrence and recurrence are indicated in *grey*. The distribution of mutations is strand asymmetric, with significantly increased C > T burden (> 10 times expected frequency) on the H-strand, and significantly increased T > C burden (~ 2.5 times expected frequency) on the L-strand. These differences are likely due to differing replicative modes of the two strands (Fig. 2). Mutation distribution is for illustrative purposes only. Based on data from [29]

Recent data cast doubt specifically on the role of oxidative stress in driving mtDNA mutation. The proximal radical oxygen species generated by the respiratory chain, superoxide (O_2_^•-^), is not an efficient DNA modifier [37–39]. However, in the presence of ferric iron, O_2_^•-^ can, through Haber-Weiss and Fenton chemistry, yield hydroxyl radicals (^•^OH) that readily react with, among essentially any organic molecule, DNA bases (Fig. 3a). An intriguing series of experiments with mitochondria-specific murine knockouts of DNA repair glycosylases OGG1 and MUTYH, necessary for excision of the most common oxidised base derivative, 8-oxo-guanine, demonstrate an unaltered mtDNA mutation load when compared with controls [40]. Mitochondrial superoxide dismutase (SOD2) catalyses the conversion of O_2_^•-^ to hydrogen peroxide (H_2_O_2_), which is reactive with nucleic acid [41] but readily diffuses out of mitochondria, unlike O_2_^•-^. A further, compelling experiment assessing crosses of OGG1 knockout mice with SOD2 knockout mice did not demonstrate enhanced mtDNA mutation burden in either SOD2 mice alone or double knockouts (Fig. 3b) [40]. Overall, these experiments indicate that oxidative damage might not be a major source of mtDNA mutations, as initially believed.

As oxidative damage to mtDNA appears not to provide an adequate explanation for observed mutagenesis, an obvious next candidate would be replicative polymerase error. However, the processive fidelity of Pol γ is among the best of known polymerases from all domains of life [42]. As such, the argument for polymerase error in mtDNA mutagenesis, particularly in the absence of oxidative damage-induced mutation, is difficult to reconcile with the enhanced rate of mutation acquisition observed in mitochondria generally, beyond the cancer context [30].

Interestingly, mtDNA mutations found in human cancers display a strand-asymmetric mutational signature. Such an observation may indicate that the strand-specific mode of mtDNA replication (Fig. 2), rather than polymerase error itself, is a likely explanation of mutagenesis**.**

## mtDNA mutations in cancer

Anecdotal reports on the presence of mtDNA mutations in excised tumours have featured in the cancer literature for several decades (for detailed reviews see [9, 43, 44]. Yet, mitochondrial genetics in cancer has been largely neglected, due in part to the attention paid to nuclear DNA but also to technical issues that surrounded accurate measurement of mtDNA mutations. For instance, it was observed that many of the variants present in cancer samples were related to mitochondrial haplogroups rather than genuine mutations and were, therefore, indicative of sample contamination [45]. Further, it was proposed that detected DNA sequences presumed to be mtDNA are instead nucleus-embedded mitochondrial sequences (NUMTs), portions of mtDNA transferred to the nuclear genome during evolution. The increased chromosomal instability in tumours might lead to an increase in NUMT abundance, which could be inadvertently detected as true mtDNA mutations (discussed in [46]). These experimental issues persisted until recently, when the availability of larger datasets, such as the International Cancer Genome Consortium (ICGC) and the Cancer Genome Atlas (TCGA), and better analytical approaches demonstrated that approximately 60% of all solid tumours bear at least one mtDNA mutation [47–49]. The vast majority of these mutations are C > T and T > C transitions, present in strand asymmetric proportion across the H and L strands, respectively (Fig. 3c), likely due to the differing replicative modes of these strands (Fig. 2), and do not fit mutational patterns associated with oxidative damage (Fig. 3b) [48, 49]. Mutations and heteroplasmies that would otherwise be cleared through purifying selection occur throughout the mitochondrial genome in these cancers, with notably increased incidence in the NCR, *ND5* and a broad region containing *ND2*, O_L_, and several tRNA genes (Fig. 3b). The nature of these mutations, whether profoundly deleterious (nonsense and frameshift mutations) or less severe (majority of missense mutations and mutations in non-protein coding regions), is broadly consistent with their prevalence and abundance; severe mutations are less common and demonstrate a trend towards purifying selection, whereas regulatory region variance is more common and subject to positive selection [49]. A substantial proportion of mutations are at high levels of heteroplasmy (> 50% mutant load), with a minority (~ 8% of tumours) achieving near-complete mutation homoplasmy. As a significant proportion of these mutations are potentially pathogenic, these results indicate that primary dysregulation of mitochondrial function via mtDNA mutation is a pervasive feature of cancer. They additionally imply that higher levels of heteroplasmy or homoplasmy (and, therefore, diminished mitochondrial function) might be detrimental for cancer, corroborating the importance of some key mitochondrial functions for cancer cell survival and proliferation that are augmented by partial mitochondrial dysfunction. This notion is supported by the finding that, in general, oncocytic tumours harbouring mtDNA mutations at high heteroplasmy (with significant mitochondrial dysfunction) are benign, non-aggressive, low proliferating lesions [50, 51]. Similarly, renal oncocytoma, characterised by defects in complex I, exhibit clear mitochondrial and metabolic defects that are a barrier to tumorigenesis [52, 53].

Beyond mutation alone, cancer-specific alterations in mtDNA copy number, either specific downregulation or upregulation [54], with similar variations at the mtRNA level [49, 55] have also been described, potentially corroborating the increased mutation abundance within regulatory regions [49]. These data appear to support the hypothesis that mitochondrial genetic defects and metabolic plasticity comprise the basis for cancer-specific metabolic rewiring strategies that encourage tumour initiation and progression [9].

## mtDNA mutations: driver, backseat driver, or passenger?

Several lines of evidence indicate that dysregulation of mitochondrial function plays an important role in cancer biology, and this has been discussed in recent seminal reviews (see for instance [8, 56]). Robust experimental evidence for a causative, cancer-driving role of mtDNA mutations has, however, remained elusive. Experimental approaches to determining a role for mtDNA mutations in cancer-associated mitochondrial dysfunction have yet to yield conclusive data, mostly because of the genetic intractability of the mitochondrial genome and consequently limited experimental tools [57]. Despite this major technical hurdle, compelling data exist that hint at the nature of mtDNA-linked mitochondrial dysfunction in cancer.

Focused clinical studies of mtDNA mutations in stratified patient cohorts have been reported in the recent past. One such report, in prostate cancer patients, demonstrated a synergistic or phenotype-modifying effect (if not true driving effect) of mtDNA mutations in the NCR on prostate cancer aggression [58]. Further, a study of Hürthle cell carcinoma patients revealed recurrent homoplasmic and near-homoplasmic mutation of various mtDNA-encoded complex I genes, associated with widespread chromosomal loss, in nearly half of the cohort [52]. The mutations of mtDNA detected were present in primary, recurrent, and metastatic tumours, suggesting a true driver role for mtDNA mutations in thyroid cancer. Unavoidably, however, the nature of such clinical data, although suggestive of a role for mtDNA mutations in cancer, cannot be used to infer their causative role.

Nuclear transfer experiments, where nuclei of cancer cells bearing mtDNA mutations and non-cancerous healthy cells without mtDNA mutations are exchanged, demonstrate that a cancer cell nucleus does not transform the enucleated healthy cell cytoplasm, and instead results in an apparently healthy cell without abnormal morphology, proliferation, or migration properties. However, transfer of the healthy nucleus into enucleated cancer cytoplasm, bearing mtDNA mutations, results in a pro-metastatic transformation [59]. Many further variations of this experiment using different cell types have produced comparable results, implicating mitochondrial dysfunction in carcinogenesis (for a review of the field, see [60]). A conceptually similar study, using MNX transmitochondrial polyoma virus middle T-driven mouse strains of breast cancer, demonstrated significant changes in tumorigenicity and metastatic potential when non-pathogenic mtDNAs are switched between the nuclear backgrounds of mouse strains [61]. Such a switching of inbred strain-specific mtDNA haplotypes between nuclear backgrounds is likely to alter respiratory fitness due to co-evolution of nuclear and mitochondrial components of the respiratory chain [62], thus crudely mimicking a pathogenic mtDNA. This effect was later shown to vary, dependent upon oncogenic driver mutations, demonstrating the potentially inconsistent impact of mtDNA variants in cancer [63]. Overall, whilst providing intriguing preliminary data, such experiments are simplistic, easily criticised, and fail to provide mechanistic insight.

**Fig. 4.**
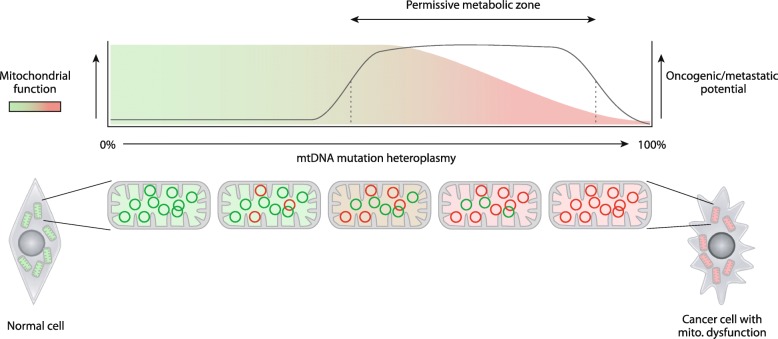
A model for ‘oncogenic/metastatic licensing’ through mtDNA mutation-derived mitochondrial dysfunction. Although mitochondrial dysfunction can be advantageous to cancer cells, and possibly oncogenic to normal cells, total ablation of mitochondrial function is likely detrimental to both. The genetic and metabolic plasticity afforded to cells bearing heteroplasmic mutations permits greater oncogenic/metastatic potential once a threshold for heteroplasmy-induced mitochondrial dysfunction is reached. A ‘permissive metabolic zone’ of heteroplasmy-induced mitochondrial dysfunction is proposed. *Green circles*, wild-type mtDNA; *red circles*, mutant mtDNA

A recent study from the authors’ laboratories more directly addresses the question of the role of mtDNA-linked mitochondrial dysfunction in cancer cells, using an osteosarcoma cell line bearing the known pathogenic mtDNA variant m.8993 T > G. This mutation leads to an amino acid change in a key, proton-translocating subunit of ATP synthase, resulting in mitochondrial dysfunction at high levels of heteroplasmy [64]. Taking this initial cell line bearing ~ 80% m.8993 T > G and using newly developed mtDNA engineering tools, mitochondrially targeted zinc-finger nucleases (mtZFN), to finely manipulate or ‘tune’ the heteroplasmic mutation load in a directed manner towards wild type [65–67], it was possible to produce a collection of isogenic cancer cell lines that varied only in mtDNA mutation load, known as mTUNE. Analysis of mTUNE cells confirmed that mitochondrial dysfunction related to m.8993 T > G supports a pro-glycolytic metabolic program that drives cell proliferation and migration, phenomena that are lost when the mutation load is reduced [68]. mTUNE additionally enabled us to describe a new connection between cytosolic reductive carboxylation of glutamine, a phenomenon frequently observed in cells with mitochondrial dysfunction, and glycolysis, which are biochemically coupled by Malate dehydrogenase 1 (MDH1) for the supply of reducing equivalents. Such exciting findings offer support to the emerging vision of mtDNA mutations acting to modulate oncogenic properties of cancer cells, causing an oncogenic or metastatic metabolic switch (Fig. 4). However, substantial further data are required to fully establish the mechanisms underpinning this link.

## Outlook

Metabolic dysfunction is a principal component of cancer. From studies of primary mitochondrial disease, it is clear that mutations of the mitochondrial genome can lead to profound metabolic deficiency [46], and from large-scale analysis of ICGC and TCGA datasets it is clear that mtDNA mutations are a very common occurrence across all solid cancers [45–47]. While recent, focused clinical and genetic studies offer a view of mtDNA mutations as potential drivers or phenotypic modifiers of prostate and thyroid cancers [52, 58], robust experimental evidence in support of a role for mtDNA mutations in cancer is lacking.

A unified mechanism describing the role of mitochondrial genetic defects in cancer initiation and progression is unlikely to be forthcoming, most probably because the metabolic flexibility of mitochondria, and the variable bioenergetic outcomes mtDNA mutations can produce, allow a range of cellular strategies for proliferation and migration. We would tentatively propose a scenario where cancer cells, during tumour initiation and progression, co-opt a specific degree of mitochondrial dysfunction that depends on their bioenergetic needs and nutrient availability (Fig. 4). Beyond permitting a metabolic switch that might favour anabolism, the dysregulation of mitochondrial function might also provide substrates that support (epi)genetic changes, which can drive or fine-tune oncogenic properties. For instance, genetic silencing or ablation of a nuclear-encoded subunit of complex I in neural progenitor cells is sufficient to cause cellular transformation through mutation of p53 [69]. Also, the aberrant accumulation of fumarate, 2HG, or succinate due to primary or secondary mitochondrial dysfunction can drive epigenetic changes that support an epithelial to mesenchymal transition [70], a process known to drive cancer metastasis. As such, mitochondrial dysfunction may act as a ‘metastatic license’, rather than an oncogenic one. A similar conclusion could be drawn from the first robust experiments determining the effects of mtDNA mutations in cancer cells [68], where mitochondrial dysfunction permits NAD^+^/NADH ratio changes that favour increased glycolysis, cell proliferation, and migration. Clinical data on the role of mtDNA mutations in prostate cancer aggression and thyroid cancer progression would also seem to agree with this concept [52, 58]. At the same time, however, mtDNA mutations can have a detrimental effect on the cancer cell. For instance, severe defects in complex I are known to diminish levels of NAD^+^ required by aKG dehydrogenase, leading to an increase in the aKG:succinate ratio, overactivation of prolyl hydroxylases, and the eventual destabilisation of hypoxia inducible factors (HIF), even at low oxygen tension, reducing tumour indolence [71]. Interestingly, complex I-deficient tumours exhibit normal angiogenesis, despite their inability to stabilize HIFs, likely due to the contribution of cancer-associated macrophages activated by a non-cell-autonomous mechanism [72].

At present, mutations of mtDNA seem likely to provide cancer cells with additional routes to tumour initiation and progression, although profound mtDNA mutation-induced mitochondrial dysfunction appears detrimental (Fig. 4). Whether such a hypothesis will persist as this young field develops remains to be seen.

## Data Availability

Not applicable.
